# Hepatitis, testicular degeneration, and ataxia in DIDO3-deficient mice with altered mRNA processing

**DOI:** 10.1186/s13578-022-00804-8

**Published:** 2022-06-07

**Authors:** Julio Gutiérrez, Karel H. M. van Wely, Carlos Martínez-A

**Affiliations:** Department of Immunology and Oncology, Centro Nacional de Biotecnología-CSIC, Darwin 3, 28049 Madrid, Spain

**Keywords:** Dido, Hepatitis, Male sterility, Ataxia, RNA splicing, Readthrough

## Abstract

**Background:**

mRNA processing is an essential step of gene expression; its malfunction can lead to different degrees of physiological disorder from subclinical disease to death. We previously identified *Dido1* as a stemness marker and a gene involved in embryonic stem cell differentiation. DIDO3, the largest protein encoded by the *Dido1* gene, is necessary for accurate mRNA splicing and correct transcription termination. The deletion of *Dido1* exon16, which encodes the carboxy-terminal half of DIDO3, results in early embryonic lethality in mouse.

**Results:**

We obtained mice bearing a Cre-LoxP conditional version of that deletion and studied the effects of inducing it ubiquitously in adult stages. DIDO3-deficient mice survive the deletion but suffer mild hepatitis, testicular degeneration, and progressive ataxia, in association with systemic alterations in mRNA splicing and transcriptional readthrough.

**Conclusions:**

These results offer insight into the distinct vulnerabilities in mouse organs following impairment of the mRNA processing machinery, and could aid understanding of human health dependence on accurate mRNA metabolism.

**Supplementary Information:**

The online version contains supplementary material available at 10.1186/s13578-022-00804-8.

## Background

Exon splicing, transcription termination, and additional processing by pre-mRNA cleavage and polyadenylation are fundamental steps in the expression of vertebrate genes. These processes are intricately coupled and rely on complex molecular machinery, many components of which are still poorly known [1, 2]. Malfunction of these components can result in misexpression of genes and might lead to disease [3–6]; it is estimated that ~ 15% of human hereditary pathologies and cancers are associated with impaired alternative splicing of mRNA [7]. Moreover, mRNA processing can differ in distinct tissues and developmental stages, such that these can be differentially susceptible to disease following malfunction of the processing machinery.

Developing embryos make extensive use of alternative mRNA splicing [8]; the majority of loss-of-function mutations in splicing factors thus result in embryonic arrest early in mouse development [9]. In adult stages, the brain is regarded as the organ with the most complex alternative splicing pattern [10], which corresponds to the high frequency of abnormal mRNA processing events reported as underlying molecular causes of neurological disorders in humans [11]. Liver and testis also often show pathologies associated with disrupted mRNA processing patterns [12–14]. Symptomatic treatment is currently the only available palliation for patients suffering this type of disease. There is an ongoing effort to treat the causes of these diseases by developing drugs able to modify alternative splicing [15–17]. Our limited understanding of the mechanisms that govern mRNA processing in living tissues nonetheless hampers the progress of this effort. Better understanding of RNA metabolism and of the pathological outcomes produced by its impairment in live animals could help to develop better diagnostic and therapeutic tools for a range of human diseases.

The *Death-inducer obliterator 1* (*Dido1*) gene is evolutionarily conserved among vertebrates; it is expressed in all tissues, with higher levels in embryonic stem cells (ESC) [18]. It is organized in 16 exons and encodes three protein isoforms of increasing size, DIDO1, DIDO2, and DIDO3. The principal isoform is DIDO3, a nuclear protein that comprises an amino-terminal part (1176 amino acids) largely equivalent to DIDO2, plus a specific carboxy-terminal part (1080 amino acids) encoded entirely in the last and longest exon of the *Dido1* gene, E16 [19]. This DIDO3-specific protein part comprises a coiled-coil domain and a large unstructured region with Pro and Arg-rich features at the C-terminal end, reminiscent of some mRNA processing factors [20]. DIDO3 sequence tags are found recurrently in proteomic studies of components of the mRNA splicing and polyadenylation machinery [21], as well as in catalogs of RNA-binding proteins [22]. Its interactions with the splicing factor SFPQ [23] and the RNA/DNA helicase DHX9 [24] directly implicate DIDO3 in mRNA processing. These interactions depend on the DIDO3 carboxy-terminal half, whose truncation by deletion of *Dido1* E16 significantly alters mRNA processing patterns in cultured cells [23]; this same deletion results in embryonic arrest early in murine development [24].

To study the functional significance of DIDO3 in vivo, we used the Cre-LoxP system to generate mice bearing a conditional *Dido1* E16 deletion. We induced the ubiquitous deletion of this exon in adult mice and studied their phenotype; while the mice survive *Dido1* E16 deletion, they show transient hepatitis, permanent testicular degeneration, and progressive ataxia, in association with altered patterns of mRNA splicing, cleavage and polyadenylation.

## Results

### DIDO3 deficiency triggers growth arrest and hepatitis in mice

**Fig. 1:**
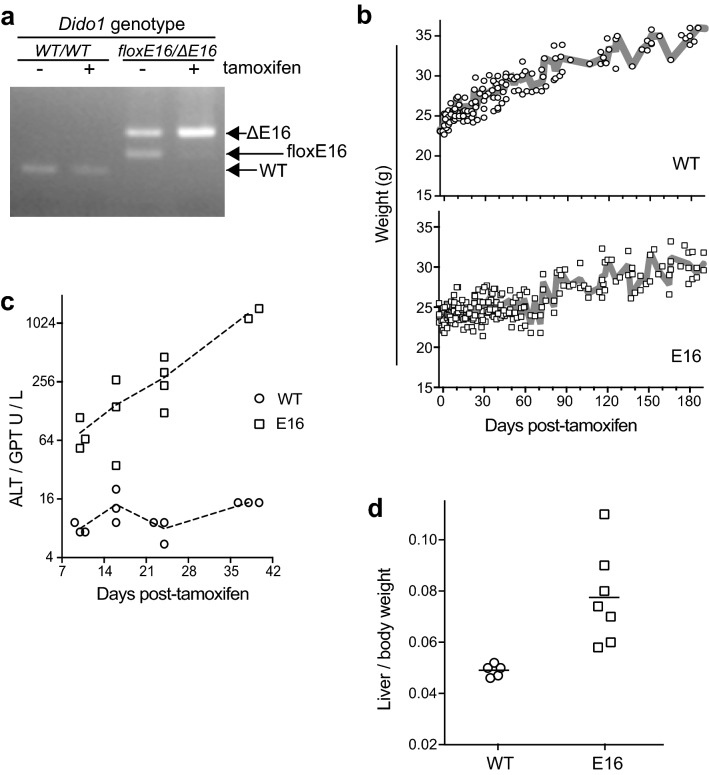
Deletion of the floxed exon and growth arrest in E16 mice. **a** Ethidium bromide-stained agarose gel showing the products of PCR analysis of genomic DNA from livers of representative *Dido1 wt/wt* or *floxE16/ΔE16* mice, untreated or tamoxifen-treated, with a combination of primers capable of amplifying all three *Dido1* alleles of interest. Results were similar for DNA from other tissues. **b** Variation in body weight over time for individual mice, plotted for each *Dido1* genotype. WT n = 11; E16 n = 21. Not all mice were weighed at every time point. Thick grey lines connect mean values. **c** Early time course of alanine aminotransferase activity in serum samples from *Dido1* WT (n = 12) and E16 (n = 12) mice. Each mouse was bled only once. Individual values are shown, as well as dashed lines connecting mean values. Aspartate aminotransferase activity followed a similar course. **d** Liver-to-body weight ratios for individual *Dido1* WT (n = 5) and E16 (n = 7) mice at 40–50 days post-tamoxifen. Bars = mean ratios; an unpaired t-test showed significant difference (p < 0.05)

Whereas the constitutive *Dido1* ΔE16 mutation is lethal in homozygosis in mouse embryonic stages, its potential effects in adult tissues were unknown. To study the functional significance of DIDO3 in vivo, E16 was deleted in adult mouse tissues (see Methods). Preliminary analysis of DNA from tamoxifen-injected mice showed that *Dido1 floxE16/*Δ*E16* tissues were efficiently converted to Δ*E16/*Δ*E16* (Fig. 1a)*.* For simplicity, the tamoxifen-treated *Dido1 floxE16/*Δ*E16* mice are referred to hereafter as E16 mice.

At the time of deletion induction, WT and E16 mice were growing similarly. WT mice continued to gain weight steadily, whereas E16 stopped growing for ~ 70 days; they then resumed weight gain, although they never reached WT levels (Fig. 1b).

The temporal reduction in E16 weight gain hinted at an underlying metabolic alteration. To explore potential metabolic anomalies, we drew blood from E16 and WT mice 3–5 weeks after tamoxifen treatment; samples were allowed to clot and centrifuged to isolate serum. Serum samples were profiled for concentrations of 12 metabolites: urea nitrogen, total protein, lipase, alanine and aspartate aminotransferases, calcium, creatinine, alkaline phosphatase, glucose, total bilirubin, phosphorus and albumin. Alanine and aspartate aminotransferase enzymatic activities were increased in E16 samples compared to WT. A kinetic study showed that E16 aminotransferase activities were above WT values by day 10 post-tamoxifen and continued to increase up to day 40 (Fig. 1c). Around day 43, free cholesterol values in E16 mice were also higher than in WT (43.9 mg/dl E16, SD 7.1, n = 6 *vs*. 23.0 mg/dl WT, SD 9.3, n = 6, p < 0.04). A cohort of WT and E16 mice was sacrificed at days 40–50 post-tamoxifen; necropsy showed that E16 livers were enlarged relative to WT (Fig. 1d).

**Fig. 2:**
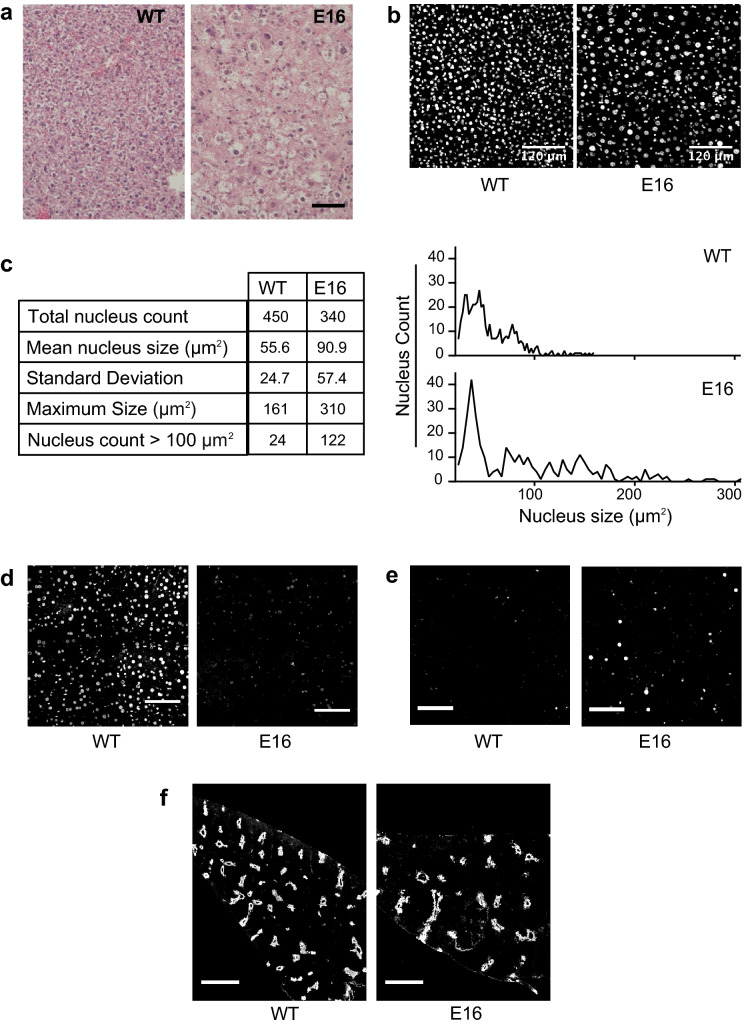
Histopathology of livers from E16 mice in acute phase.** a** Hematoxylin/eosin-stained sections of liver samples from representative WT and E16 mice. Bar = 100 μm. **b** Cell nuclei in DAPI-stained liver sections. **c** Nuclei counted in the micrographs in b and their distribution according to size, in WT *vs*. E16 liver samples. Mean nuclear size differs between genotypes, as assessed by unpaired t-test (p < 0.05). **d** Anti-DIDO3 fluorescent staining of liver sections in stitched 2 × 2 confocal tiles. Bar = 100 μm. **e** Anti-PCNA fluorescent staining of liver sections. Bar = 100 μm. **f** Anti-glutamine synthetase fluorescent staining of liver sections. Bar = 800 μm

Liver samples were then evaluated by histopathology. Hematoxylin/eosin staining revealed mild to moderate hepatitis in E16 sections (Fig. 2a), with ballooning degeneration in the parenchyma and occasional foci of lymphocytic infiltration. In addition, E16 hepatic cell sizes appeared larger than WT. The number of nuclei per microscopic field was reduced in DAPI-stained E16 sections examined by fluorescence microscopy (Fig. 2b, c left). Many E16 nuclei were also abnormally large (Fig. 2c). Liver sections were immunostained with a DIDO3-specific antibody and examined by fluorescence microscopy. WT samples showed widespread nuclear staining, whereas the vast majority of E16 hepatic cells were not stained (Fig. 2d); this corroborated that tamoxifen treatment had efficiently excised *Dido1* exon 16 in liver. Additional immunostaining of liver sections showed increased expression of the DNA replication marker PCNA in nuclei of E16 samples (Fig. 2e). To determine whether the functional zonation of the basic liver lobules was affected in E16 mice, liver sections were also immunostained with anti-glutamine synthetase, a marker of pericentral zonation. Stained sections examined at low power showed slightly reduced lobule density in E16 mice, although overall pericentral zonation was conserved (Fig. 2f).

Beyond day 43 post-tamoxifen, transaminase levels decreased progressively in E16 mouse sera, reaching values near WT by day 114 (Additional file 1a); liver-to-body weight ratios also normalized to ~ 0.05 in E16 mice by that time. In agreement, liver samples from these E16 mice showed only minor signs of hepatitis (Additional file 1b); they also showed broad DIDO3 immunostaining (Additional file 1c), in contrast to the samples from the peak of hepatitis.

Occasional tumors were detected in livers of both E16 and WT old mice (≥ 1 year), which were diagnosed by histopathological analysis as adenomas and hepatocarcinomas. Both the tumors and healthy tissue in WT mice were DIDO3-positive; in contrast, the tumors in E16 mice were DIDO3-negative, within mostly DIDO3-positive healthy tissue (Additional file 1d).

Other organs and tissues were inspected visually, weighed, and subjected to histopathological analysis, with no significant findings except for testis (Additional file 2).

### Testis degeneration and infertility in mice lacking DIDO3

**Fig. 3:**
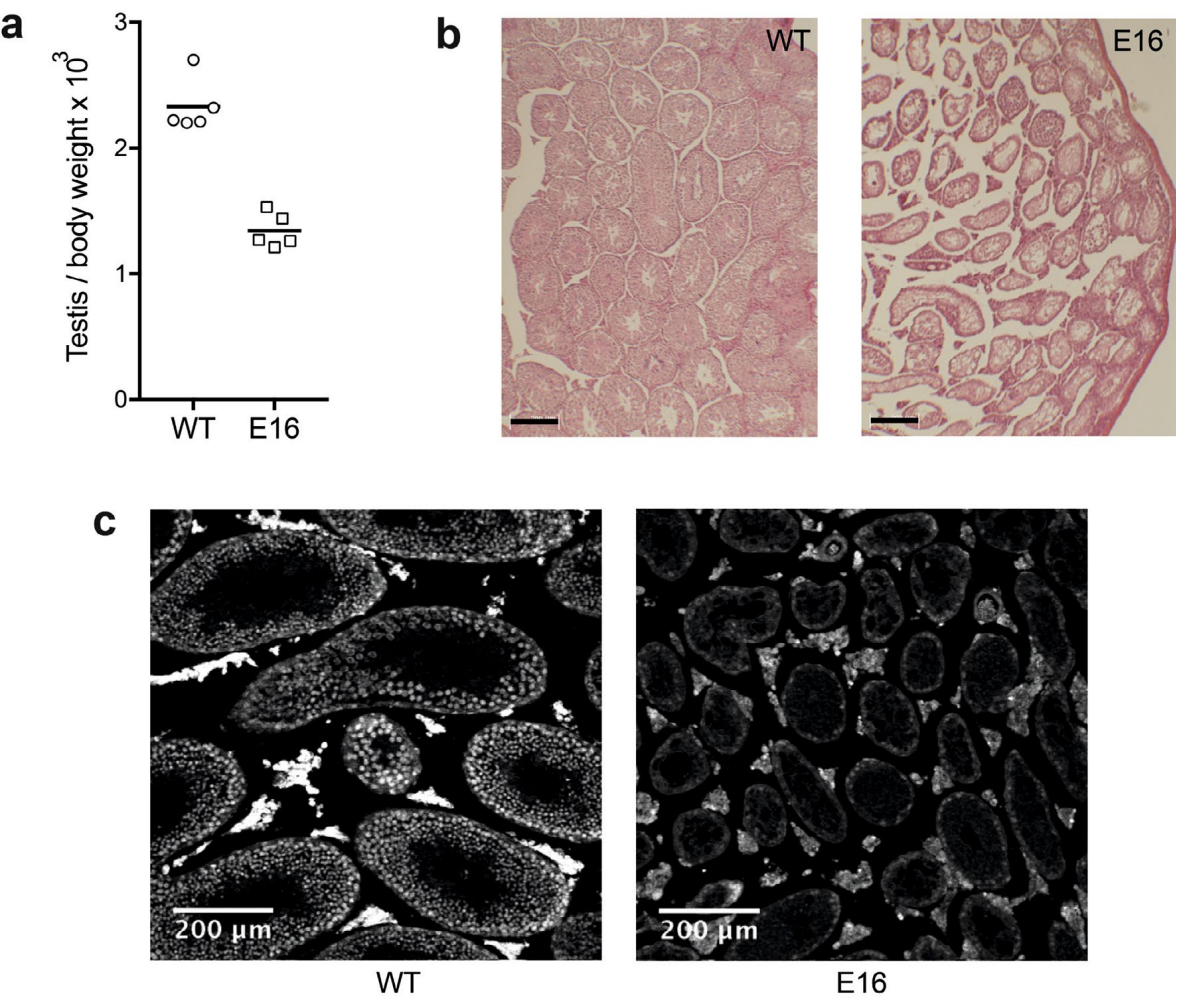
Testis degeneration in E16 mice. **a** Testis-to-body weight ratios for individual *Dido1* WT (n = 5) and E16 (n = 5) mice. Bars are mean ratios; an unpaired t-test showed significant difference (p < 0.05). **b** Hematoxylin/eosin-stained sections of testis samples from representative WT and E16 mice. Bar = 200 μm. **c** Anti-DIDO3 fluorescent staining of testis sections

In addition to decreased body weight, visual inspection of live E16 males suggested testis hypoplasia. By day 35 post-tamoxifen and thereafter, necropsies of E16 male mice confirmed decreased testes size and testis-to-body weight ratio (Fig. 3a). Histopathologic analysis of E16 testes samples showed degeneration, with partial collapse of seminiferous tubules and defective germ line maturation (Fig. 3b). As for liver, immunostaining confirmed extensive loss of DIDO3 protein in E16 testes (Fig. 3c).

Mature E16 *vs*. WT male mice were mated with WT females. Of five WT x WT crosses, one was infertile, whereas four of seven E16 x WT crosses were infertile, in rough agreement with the testicular defects observed in mutant males.

### DIDO3 deficiency induces ataxia in mice

Young E16 mice showed less movement inside the cage than their WT counterparts. They also looked less steady when placed on the wires, and some showed hind limb-clasping behavior when lifted by the tail. These traits increased progressively with aging until about 1 year of age, when some E16 mice had epileptic seizures and many showed a noticeable movement disorder, most apparent as staggering gait and trembling. An additional movie file shows these traits in more detail [see Additional file 3]. The combination of these observations suggested neuromotor deficiencies in E16 mice, specifically ataxia.

**Fig. 4:**
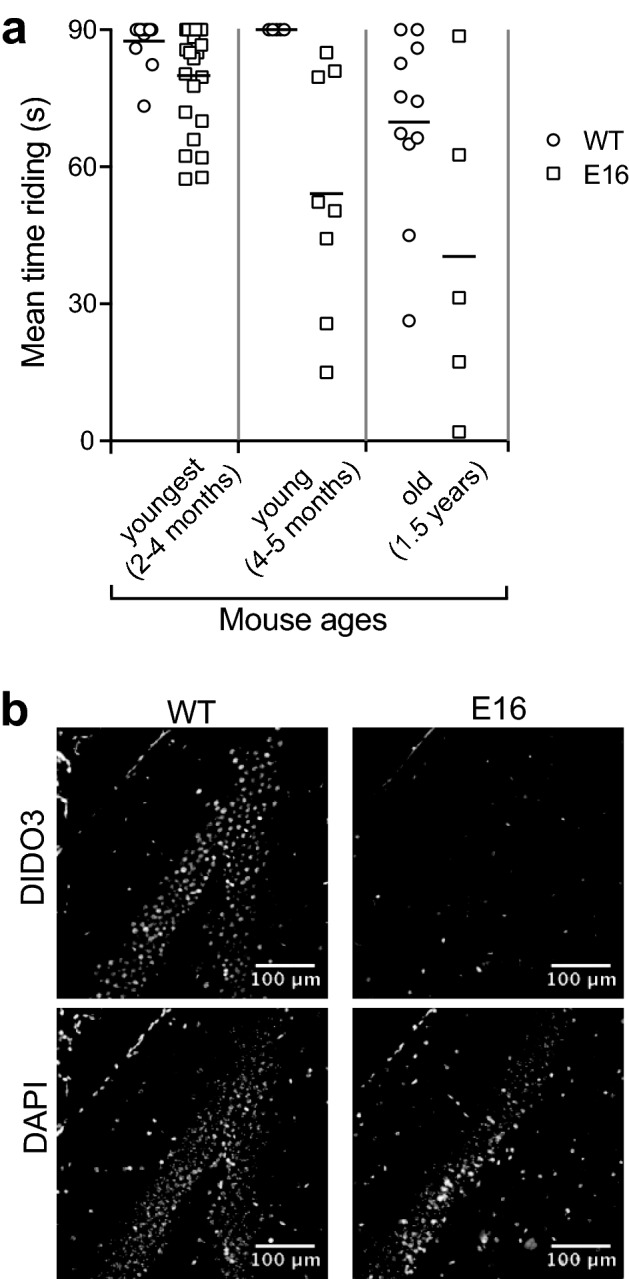
Ataxia in E16 mice.** a** Performance of *Dido1* WT and E16 mice on the rotarod, stratified by age. Each dot represents the mean score of three independent trials for each mouse. Youngest cohort WT n = 12, E16 n = 24; young cohort WT n = 5, E16 n = 8; old cohort WT n = 11, E16 n = 5. Bars are group means; an unpaired t-test showed significant difference (p < 0.05) at all three ages. **b** Anti-DIDO3 fluorescent staining of representative brain sections showing the dentate gyrus. The DAPI channel shows similarly abundant nuclei in *Dido1* WT and E16 samples

To analyze their neuromotor function quantitatively, we assessed WT and E16 mice in rotarod tests. The youngest E16 mice tested (~ 3 months of age) already showed a tendency to impaired performance on the rotarod; this could be influenced by the liver disease. By 4–5 months, well after the hepatitis peak, all WT mice tested rode for the full 90 s of the test, whereas none of the E16 mice reached that score; most fell before 50 s. Finally, most old mice (~ 1.5 years) performed poorly on the rotarod compared to young mice of the same genotype, but the average score for old E16 mice was clearly poorer than that of old WT mice (Fig. 4a).

DIDO3 immunostaining in brain was examined by fluorescence microscopy. As for other organs, E16 brains showed a radical decrease in DIDO3 expression compared to WT (Fig. 4b), which indicated that tamoxifen had crossed the blood–brain barrier and efficiently induced *Dido1* E16 deletion. Staining results were similar for samples from young and old mice.

### Altered mRNA processing in DIDO3-deficient mice

To better understand the molecular mechanisms that underlie the physiological defects observed in E16 mice, we analyzed RNA samples from WT *vs.* E16 livers by deep sequencing. To capture driver molecular events rather than hepatitis side effects, samples were obtained from mice 10–20 days before the peak of the disease.

After alignment of the qualified reads to the mouse genome reference, we detected the expression of 15500 genes, on average (cut-off > 0.025 transcripts per million). Most genes were expressed at similar levels in WT and E16 samples; however, E16 overexpressed an average of 102 genes and underexpressed 153 compared to WT (fold change > 2, false discovery rate [FDR]-adjusted p < 0.05; Additional file 4).

**Fig. 5:**
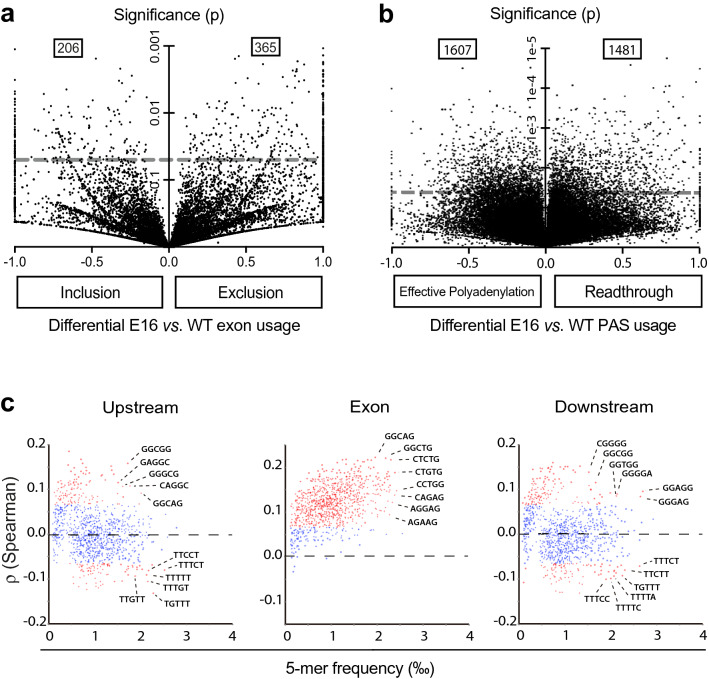
Abnormal mRNA processing in E16 livers. **a** Volcano plot of exon exclusion or inclusion events in RNA-seq data, from Additional file 5. Each dot represents a single exon. They are distributed across the x-axis according to a value between 0 and + 1 or -1 calculated for each exon as a measure of its mean exclusion or inclusion frequency in E16 compared to WT livers, and across the y-axis according to the p value for the difference between mean usage by E16 *vs*. WT livers, calculated by Welch's t-test. Dots stacked at the right edge represent exons skipped in E16 to some extent, but not at all in WT; dots at the left edge represent exons included in E16, but not in WT. The grey dashed line marks the p = 0.05 level. Many dots accumulate below this line, near the axis origins, indicative of abnormal exon usage both quantitatively small and also inconsistent. The 571 dots above the dashed line identify significant (p < 0.05) events of abnormal exon usage (206 inclusion, 365 exclusion) in mRNA from E16 livers. **b** Volcano plot of polyadenylation site (PAS) usage in RNA-seq data from Additional file 6. Each dot represents a single potential PAS. Values are represented as in panel a. The 3088 dots above the dashed line identify significant (p < 0.05) events (1607 effective PAS usage, 1481 transcription readthrough). **c** Correlation between abnormal exon usage and base composition biases in E16 livers. Each dot represents a single 5-mer motif; they are distributed across the x-axis according to their frequency of occurrence upstream (left), inside (middle), or downstream (right) exon boundaries, and across the y-axis based on their Spearman correlation with corresponding exon usage. Positive correlation indicates increased exon exclusion in the E16 mutant, relative to WT, whereas negative correlation indicates increased exon inclusion. Motifs showing significant correlation (n = 571, ρ > 0.0655 for positive correlation, ρ < − 0.0655 for negative correlation) are highlighted in red. Sequences of example motifs with statistically significant correlation and frequent occurrence are indicated

The aligned reads were analyzed for exonic regions usage. The results showed that 571 exonic RNA regions, corresponding to 488 different gene bodies, were abnormally spliced in E16 livers; of these, 365 exons were excluded relative to their usage in WT livers, whereas 206 were included (p < 0.05; Fig. 5a; Additional file 5).

By extending the analysis to reads mapped downstream of the nominal 3’ end of each gene body, the RNA-seq data were also scanned for potential transcriptional readthrough in E16. The results showed 1481 events of abnormal transcription prolongation in E16 *vs* WT livers (p < 0.05; Fig. 5b; Additional file 6).

A previous study of mouse embryonic fibroblasts (MEF) bearing an E16 mutation on *Dido1* showed that base compositional biases around exons strongly influence their inclusion or exclusion in mRNA splicing [23, 25]. To determine whether there is a similar effect in E16 livers, we carried out a dedicated computational analysis on our RNA-seq data. In agreement with the observations for MEF, we found that abnormal exclusion of exonic mRNA regions in E16 livers relative to their use in WT correlated with the presence of G-rich motifs in surrounding sequences, whereas abnormal exonic inclusion correlated with T-richness (Fig. 5c; Additional file 7).

**Fig. 6:**
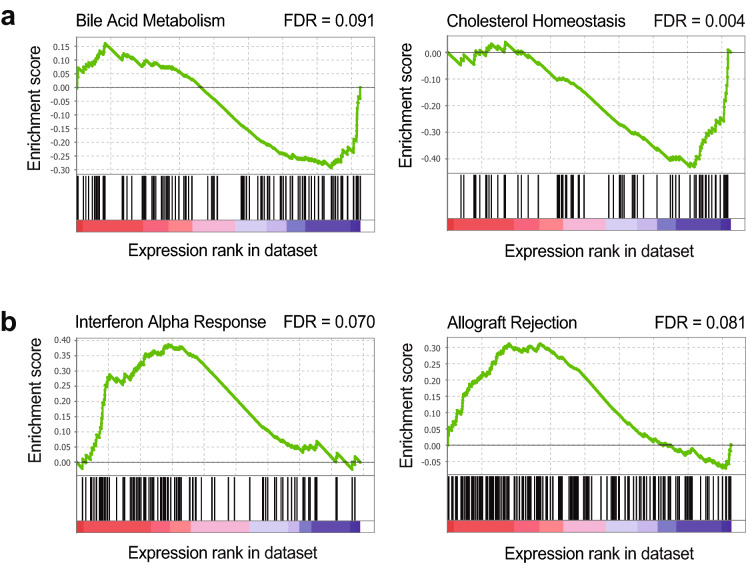
Hallmark pathways in E16 livers. The GSEA program was used to identify gene ontology terms enriched or depleted in the RNA-seq dataset for genes differentially expressed in E16 *vs*. WT livers. Examples of terms with relevance to the mutant phenotype are plotted. X-axes represent relative expression level of genes in the ontology group (red, increased expression in E16; blue, decreased expression in E16). Y-axes represent gene group enrichment (> 0) or depletion (< 0) in E16. **a** Gene ontologies preferentially depleted in E16 samples include typical liver-related pathways such as Bile Acid and Cholesterol metabolism. **b** Gene ontologies preferentially enriched include terms such as IFN-α response and Allograft Rejection, consistent with liver inflammation in E16 mice

To clarify the connection between impaired mRNA processing and phenotype in E16 livers, we performed gene set enrichment analysis (GSEA) on the RNA-seq datasets. In accordance with predictions for defects in a general mechanism of gene expression, no specific gene set was significantly enriched when GSEA was applied to the differential exon usage dataset. In contrast, when applied to the raw differential gene expression dataset, GSEA yielded gene set enrichments; among others, cellular processes associated with liver function were underrepresented in E16 (Fig. 6a) whereas processes corresponding to inflammation were overrepresented (Fig. 6b). Since inflammatory infiltration was rare in most mutant samples, this result suggested that the mutant hepatocytes react to impairment in mRNA processing by triggering innate inflammatory responses.

**Fig. 7:**
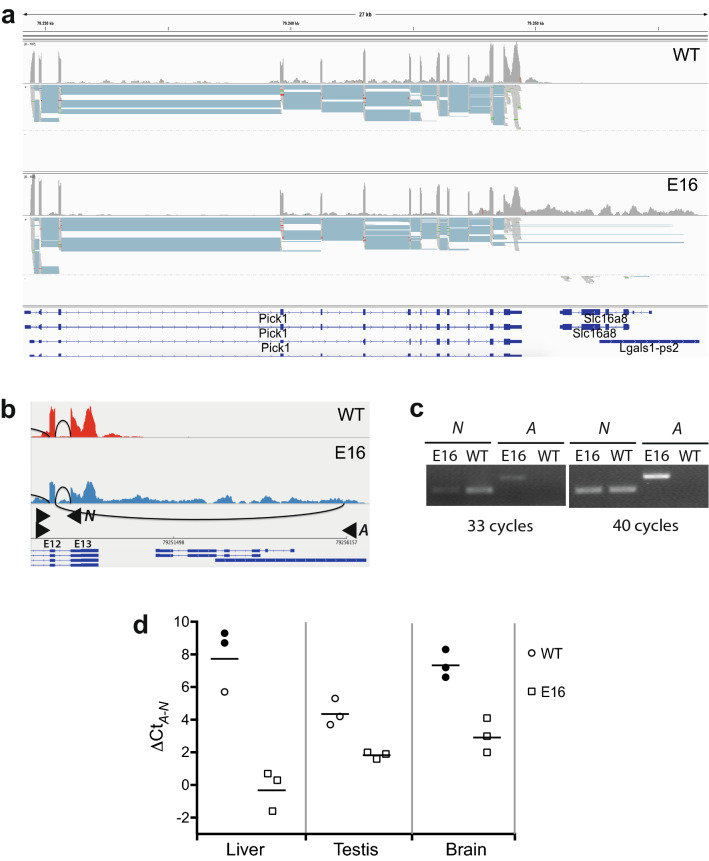
Altered *Pick1* mRNA processing in E16 tissues.** a** Coverage and alignment of RNA-seq reads from representative *Dido1* WT and E16 livers to the mm10 reference genome at the *Pick1* locus*.* The upper frame in each plot depicts coverage; blue tracks in the lower frames represent individual junctions. Note RNA transcription downstream of the last *Pick1* exon through the *Slc16a8* + *Lgals1-ps2* loci in E16.** b** Detailed view of the coverage and splice junctions at the 3' end of *Pick1* and across *Lgals1-ps2*, and scheme of the oligonucleotide primer pairs designed to assess normal (*N*) *vs*. abnormal (*A*) *Pick1* mRNA splicing. **c** Ethidium bromide-stained agarose gel showing products of the PCR analysis of cDNA from representative *Dido1* WT *vs*. E16 livers with the primer pairs depicted in b. To render this analysis semiquantitative, the PCR was run independently for 33 and 40 cycles. **d** Plot of ΔCt_*A-N*_ values from qPCR analysis of mRNA splicing at the *Pick1* locus on cDNA samples from three tissues, each from three individual *Dido1* WT *vs*. E16 mice. Filled symbols represent lower ΔCt_*A-N*_ limits in cases in which abnormal splicing was not detected. Bars are mean ΔCt_*A-N*_ for each group; an unpaired t-test showed significant difference ( p < 0.05) between E16 and WT in all three organs

The mRNA processing abnormalities observed in E16 livers are similar to those previously reported in E16 MEF, and they range evenly across the repertoire of expressed genes. Both of these findings are consistent with the idea that the processing abnormalities are a primary effect of the mutation and do not depend on the tissue in which it is expressed. To confirm this, we conducted an RT-PCR scan of RNA extracted from different tissues, targeting a sample of five genetic loci that, being good cases of abnormal RNA processing in E16 livers, also showed broad tissue expression. *Pick1*, for instance, is a 20 kb gene for which transcription in E16 liver proceeds through 7760 additional bases. *Pick1* readthrough spans a pseudogene encoded downstream in the same strand, *Lgals1-ps2*, which is silent in WT (Fig. 7a). In addition, in a fraction of these abnormal transcripts the last *Pick1* exon (E13) is ignored for splicing and the penultimate exon (E12) is spliced onto a sequence at the end of the pseudogene, which offers a good target for differential RT-PCR (Fig. 7b). Primers were thus designed to amplify the abnormal splicing event, as well as the last normal (WT) *Pick1* splicing (E12-E13) for comparison.

The *Pick1* primers were first tested in semiquantitative PCR with cDNAs from WT *vs* E16 livers as templates. Corroborating the RNA-seq data, normal splicing between the last nominal *Pick1* exons was detected in both WT and mutant samples, whereas the abnormal splicing was detected only in the mutant (Fig. 7c). The same primers were then used in qPCR with cDNAs from liver, testis, and brain from three independent WT *vs*. E16 mice as templates. The ratio of abnormal to normal splicing in each sample was calculated as the cycle threshold (Ct) corresponding to the *Pick1-Lgals1-ps2* isoform minus that of normal *Pick1* E12-E13 isoform (ΔCt_*A-N*_). For meaningful calculations and plotting, 40 was established as the Ct_*A*_ value whenever the abnormal isoform could not be detected throughout the whole PCR reaction (most WT templates). *Pick1* ΔCt_*A-N*_ was lower in all three E16 organs than in the equivalent WT organs (Fig. 7d). This indicates that *Pick1* is also mis-transcribed onto *Lgals1-ps2* in other organs affected phenotypically by the mutation, such as E16 testis and brain.

To further substantiate this analysis, we extended the qPCR study to four additional targets indicative of altered mRNA processing. Additional file 7 depicts the genetic structure of these targets as expressed in WT *vs*. E16 livers. In addition, cDNAs from three apparently healthy organs (heart, lung, duodenum) were included in the qPCR analysis in addition to those from liver, testis, and brain. The results of this multiplexed analysis on samples from three WT *vs*. three E16 mice are listed in Table 1 to show ΔCt_*A-N*_ for each target, organ, and genotype.

**Table 1:** RT-qPCR study of altered mRNA processing in different tissues of E16 mice

Organ	Target	*Dido1* genotype					
		WT (n = 3)			E16 (n = 3)		
Liver	*Gas2l1*	2.1	5.6	2.7	> 9.4	2.2	1.1
	*Osbpl2*	6.0	5.5	5.6	− 1.5	0.2	0.1
	*Ndufs7*	15.6	17.1	15.3	> 12.4	11.8	12.0
	*Wrap73*	7.2	8.9	9.1	− 0.2	1.2	1.7
Testis	*Gas2l1*	1.5	> 6.4	> 6.1	0.9	1.5	2.1
	*Osbpl2*	2.3	5.2	4.3	0.6	1.6	2.6
	*Ndufs7*	> 13	> 16.8	> 18.1	11.5	9.7	17.0
	*Wrap73*	6.2	7.7	7.9	2.9	3.0	3.5
Brain	*Gas2l1*	6.7	> 6.8	> 5.5	1.1	1.2	6.7
	*Osbpl2*	4.4	7.0	6.5	− 0.7	1.5	0.9
	*Ndufs7*	> 13.8	> 18.1	> 18.3	10.7	11.9	17.5
	*Wrap73*	10.1	> 13	> 12.9	3.6	3.4	5.7
Heart	*Pick1*	> 8.6	> 7.0	> 2.6	5.0	6.2	4.0
	*Gas2l1*	3.3	> 6.3	> 6	− 1.3	1.5	2.5
	*Osbpl2*	5.2	8.2	> 10.6	− 0.5	0.9	3.9
	*Ndufs7*	15.8	> 20.9	> 20.3	7.5	11.2	> 19.5
	*Wrap73*	8.5	> 7.9	> 7.7	3.6	3.7	2.3
Lung	*Pick1*	> 6.4	> 4.1	> 3.8	3.6	5.4	5.0
	*Gas2l1*	3.6	> 7.1	> 6.7	− 0.9	1.0	− 0.3
	*Osbpl2*	4.4	6.3	> 3.6	− 1.0	2.3	ND
	*Ndufs7*	13.8	> 17.4	> 14.8	8.7	10.3	> 10
	*Wrap73*	8.1	7.3	> 10.5	1.9	2.1	3.9
Duodenum	*Pick1*	> 6.0	8.3	ND	3.0	ND	1.1
	*Gas2l1*	6.2	5.6	> 3.9	7.0	− 0.4	1.7
	*Osbpl2*	2	4.4	2.0	− 1.1	− 1.2	− 0.6
	*Ndufs7*	> 11.8	> 18.6	> 16.2	> 9.7	9.1	11.3
	*Wrap73*	8.2	6.4	> 10.0	4.0	1.5	1.1
Average ΔCt_*A-N*_ (lower limit)		> 8.6			> 4.2		

Overall ΔCt_*A-N*_ values were several cycles lower in E16 mice than in WT for most targets in most organs (average ΔΔCt_*A-N*_ between WT and E16 > 4.3). Most E16 organs thus expressed many more of the abnormal transcripts than the corresponding WT, regardless of whether the organs were diseased or healthy. The E16 mutation thus produces systemic alterations of mRNA processing.

## Discussion

We previously reported a role for the DIDO3 protein in mRNA metabolism in in vitro*-*cultured cells, as well as the early embryonic lethality of mutant mice lacking *Dido1* exon 16 (E16) [23, 24]. E16 encodes the carboxy-terminal half of DIDO3, to which its interactions with various components of the mRNA splicing and polyadenylation machinery have been mapped. To study the role of DIDO3 in vivo and overcome lethality, we designed and produced mice with a conditional floxE16 mutation that can be induced in adult stages.

Here we show that tamoxifen treatment efficiently converted *Dido1* floxE16 to ΔE16 deletion in adult mice expressing Cre-ERT2. This deletion was previously found to produce replication stress, chromosome segregation defects, and DNA damage in stem cells as well as in differentiated cultured cells, all of which presumably contribute to developmental arrest in mutant mouse embryos [18, 24]. In contrast, adult E16 mice survive the deletion with relatively mild disease signs, the most visible of which is delayed growth; concomitantly, transaminase activities increase in serum. Transaminases could leak into the bloodstream due to damage to the liver or other organs affected by the deletion. The observation of liver swelling and other histopathological findings confirm that the liver is indeed injured in DIDO3-deficient mice, probably sufficient to account for their growth retardation. Hepatic cell size in E16 mice increases, which could be a primary cause of the hepatomegaly observed; cell nuclei also become enlarged, which suggests an increase in their ploidy. Both in humans and in mice, polykaryons and megalokaryons are occasionally found in healthy liver, but both increase with age as well as with diseases involving abnormal cell division [26, 27]. This observation is consistent with previous results describing abnormal division and aneuploidy in *Dido1* mutant cells cultured in vitro [18, 28]. Overall, the mutation-induced damage in DIDO3-deficient liver is limited, leaving the basic central-portal zonation of lobules unaffected, as judged by the restricted expression of glutamine synthetase around central veins.

The liver is known for its regenerative capacity [29]. Abundant expression of the DNA replication marker PCNA in DIDO3-deficient livers suggests that active cell proliferation, a sign of a regenerative response, is already underway at the peak of the mutation-driven hepatitis. In accordance, transaminase levels in serum decrease progressively in E16 mice from day 43 onward. E16 mice indeed regain weight at this time, and additional analyses at later stages confirm gradual hepatitis remission. The cellular composition of regenerated E16 livers is no longer dominated by DIDO3-deficient hepatocytes and DIDO3-proficient cells regain dominance, which indicates active proliferation of the few remaining *floxE16/*Δ*E16* hepatocytes. The gradual disappearance of *Dido1 *Δ*E16/*Δ*E16* hepatocytes from E16 livers provides conclusive evidence that DIDO3-deficient cells are impaired and cannot compete with DIDO3-proficient cells in vivo.

A few ΔE16/ΔE16 hepatocytes survive and remain in the floxE16/ΔE16-regenerated liver for a long period, as inferred from the fact that occasional DIDO3-negative adenomas and hepatocarcinomas develop in aged mutant mice. It is tempting to speculate that DIDO3 deficiency renders these hepatocytes prone to carcinogenesis; in our study, more E16 mice developed liver tumors than WT mice, but cases were too few to draw conclusions.

Testis is the only organ other than liver that shows macroscopic pathologies in DIDO3-deficient mice. In contrast to the relative enlargement of liver, E16 testes are smaller than those of WT. This phenotype may have a component of hypogonadism, as the deletion is induced at an age in which the mice are incompletely mature, but microscopic examination of E16 testes also shows signs of active degeneration in the seminiferous ducts. Since these ducts make up approximately 90% of the cell mass in the testis, their degeneration amply accounts for the size decrease in E16 testes. In agreement with these defects, E16 males show decreased fertility, with the majority of mice completely sterile.

Aside from changes in organ size, the most noticeable pathological consequence of DIDO3 insufficiency in E16 mice is a motor coordination disorder. At difference from the effects on the liver, this disorder worsens with age. As a behavioral trait, motor coordination is very sensitive to defects that affect muscles or the motor branch of the nervous system, even to subtle ones. We observed no abnormalities in E16 mouse muscle, which appears healthy in necropsy or in careful histopathological examination. Although we do not exclude the possibility that E16 mice have mild muscular impairment, we assume that the predominant component in the syndrome of ataxia and episodic seizures in these mice is impaired neurological function. More specifically, the pattern of motor coordination defects in DIDO3-deficient mice and its characteristic exacerbation over time are indicative of cerebellar alterations. We have seen hints of a decrease in Purkinje cell bodies in some E16 cerebelli, but the observations are not conclusive.

Our molecular study of the diseases driven by truncation of DIDO3 focused on the hepatitis. At the peak of hepatitis, the liver disruption could leak bile and other toxic metabolites, which could potentially complicate the disease and the underlying molecular landscape. By sampling livers at an early time relative to the hepatitis peak, we aimed to study the causes rather than the effects of the disease.

Based on previous results from cultured *Dido1* E16 mutant cells [23, 24], we anticipated an abnormal pattern of mRNA processing in E16 livers; analyses of RNA sequences from these samples indeed revealed many processing defects. Both exon skipping and abnormal exon inclusion were observed in E16 livers, with a majority of skipping events; this pattern matched that of RNA processing defects reported in cultured E16 MEF [23]. In that study, the presence of G- or T-rich motifs in the mutant mRNAs correlated with abnormal exon exclusion or inclusion, respectively. Thymidine-dependent transcription pausing was later proposed to allow the DIDO3-defective splicing machinery to recognize and use relatively weak 3' splice sites that would otherwise be ignored [25]. The present study confirms in mouse liver that the efficiency of exon usage by the splicing machinery in *Dido1* E16 mutants depends greatly on downstream thymidine abundance. A similar type of compositional bias was recently reported to control exon usage through divergent modes of splicing, either by intron definition or exon definition, in an independent system [30].

Abnormal mRNA splicing and transcriptional readthrough events were also reported in E16 ESC cultured in vitro [24]; these events were attributed to defective interaction of the truncated DIDO3 with the helicase DHX9 and an increase in R-loop accumulation at the 3' end of affected genes. The pattern of mRNA processing alterations seen in vivo thus largely matches those reported in vitro, which supports the idea that most of these alterations are a primary effect of the mutation on the mRNA processing machinery. Although direct evidence is lacking, the observation of multiorganic disease in E16 mice suggests mRNA processing defects are a common underlying cause for this complex phenotype.

mRNA processing appears to be altered similarly in all organs of E16 mice, diseased or healthy, as suggested by our RT-PCR analysis of selected markers. This observation raises the question as to why those alterations should drive some organs to disease while sparing others. Indeed, most organs in E16 mice appear to remain healthy, but they may not be entirely so. Mild symptoms might have gone unnoticed in our examinations; for instance, limitations on blood sample volume make it difficult to detect small changes in the levels of some serum metabolites. We examined a broad panel of organ samples by standard histopathology, but the diagnosis of many disorders would require specific procedures we did not undertake. Our results nonetheless show a clear disparity in the response of different E16 mouse tissues to the same molecular insult, which we propose arises from an intrinsically distinct tissue vulnerability to alterations in the quality or efficiency of mRNA processing. The two organs anatomically disrupted in E16 mice, liver and testes, and nervous system function as a whole, might be more sensitive to alterations in mRNA processing. Indeed, liver stands out for its peculiar alternative mRNA splicing, qualitatively different from most other organs, due possibly to its distinctive complement of splicing factors [31]. In addition, the liver might be especially sensitive to the accumulation of mRNA misprocessing products, which could trigger innate inflammatory sensors of abnormal RNA, ultimately resulting in the hepatitis phenotype. The enrichment in inflammation-related pathways apparent in our GSEA analysis of E16 liver RNA, in the absence of significant inflammatory infiltration, is consistent with this possibility.

Brain and testis are reported to display the most diverse mRNA splicing and polyadenylation repertoire of all organs [32–34]. In the case of testis, which itself is special with regard to the wide variety of genes it expresses [35, 36], changes in global mRNA splicing patterns are associated mainly with the switch from mitosis to meiosis [37, 38]. In contrast, stomach and intestine have the lowest levels of alternative mRNA splicing in comparative studies [39]. The apparent tolerance of the digestive tract in E16 mice to the *Dido1* mutation is compatible with this, in support of the concept that different tissues are distinctively vulnerable to perturbations in mRNA processing in mouse.

Liver diseases and azoospermia have been linked to mRNA processing defects in man [12, 40–42]. The nervous system is subject to diseases associated with altered mRNA processing as well [10]. As in E16 mice, some RNA-linked neurological disorders in humans present ataxia as a clinical symptom; moreover, many are frequently exacerbated with aging [11]. The pathologies we observe in DIDO3-deficient mice thus resemble diseases that affect humans. Defects in human DIDO3 could possibly underlie or contribute to some of these disorders, in which case E16 mice could be useful for testing drugs to treat disease-related insufficiencies of the mRNA processing machinery [15].

## Conclusions

Our findings highlight DIDO3 as an important participant in correct mRNA processing in a mouse model, and point to particular sensitivity of liver, testis, and the nervous system to the systemic impairment of this processing. This knowledge could contribute to a better understanding of the importance of accurate mRNA processing in human health and disease.

## Methods

### Mice

Founder B6J.Cg-*Dido1*^*tm3Cmar*^/J mice carrying a conditional E16-loxP-flanked allele of *Dido1* were produced by Ozgene Pty (Australia). The production of mice bearing *Dido1*^*tm3.1Cmar*^, the Cre-deleted E16 allele (ΔE16), was reported elsewhere [24]. These mutant mice and congenic *Dido1* wild type mice (WT) were bred with B6.Cg-*Ndor1*^*Tg(UBC−cre/ERT2)1Ejb*^/2 J (JAX #008085) [43] to generate experimental cohorts of *Dido1 floxE16/*Δ*E16 vs*. *Dido1* WT/WT mice, all bearing a single copy of the *cre-ERT2* transgene under the control of the human *ubiquitin C* promotor. These mice constitutively express the Cre-ERT2 fusion protein in all tissues, which renders them susceptible to full, ubiquitous deletion of their E16-floxed allele after tamoxifen injection; the heterozygous *floxE16/*Δ*E16* mice otherwise show no obvious phenotype. At the age of 6–7 weeks, each mouse cohort received daily intraperitoneal injections of tamoxifen (Sigma-Aldrich T5648) dissolved in corn oil, at a dose of 80 μg/g body weight, for 5 days. Control mice that received oil vehicle only showed no phenotype. Male and female mice were used and yielded similar results, but because of phenotypes observed in testis, most mice tested were males.

All procedures involving animal care and management were carried out at the authorized facility in the Centro Nacional de Biotecnología. B6J.Cg-*Dido1*^*tm3Cmar/tm3.1Cmar*^/J mice are deposited in the EMMA repository with ID 12287.

### Antibodies and PCR primers

To produce an antibody specific for mouse DIDO3, a histidine-tagged recombinant protein with an amino acid sequence corresponding to that encoded in an E16 segment of mouse *Dido1* was produced in *Escherichia coli*, purified, and used to immunize rabbits. Sera were obtained and tested in western blot for specific recognition of natural DIDO3 from mouse samples. The most reactive serum was selected and purified with G protein.

Rabbit polyclonal anti-PCNA ab2426 and anti-glutamine synthetase ab49873 IgG were from Abcam. ProLong Gold Antifade Mountant with DAPI (Invitrogen P36941) was used to mount fluorescently stained slides and stain nuclei. A Zeiss laser scanning confocal microscope was used to acquire images, which were processed and quantified with ImageJ [44].

Quantitative polymerase chain reactions (qPCR) were run for 40 cycles in a QuantStudio 5 device (Applied Biosystems) using HOT FIREPol DNA polymerase (Solis BioDyne 01-02-00500). The sequences of oligonucleotide primers used in PCR are provided in Additional file 9.

### Rotarod

The rotarod test quantifies neuromotor abilities of experimental mice as a composite output of balance, grip strength, and endurance. A five-lane Ugo Basile device was used in acceleration mode. Preliminary tests indicated that the best condition in which to distinguish performances across the experimental mouse cohorts was an accelerating ramp from 4 to 30 revolutions per minute (rpm) over 90 s (s). WT mice and E16 mice, blinded for genotypes, were tested in each ride. Mice were subjected to one training ride (60 s; constant 4 rpm), followed by one trial in which time latency to fall was recorded. The full 90-s score was assigned to those mice that did not fall during the entire trial. The test was repeated three times, once every other day, and the scores of each mouse were averaged for plotting.

### RNA isolation and high-throughput sequencing (RNA-seq)

Organs were promptly dissected from euthanized, exsanguinated mice and placed on ice; samples were excised and immediately frozen in liquid nitrogen. Samples were then stored at − 80 ºC until high quality total RNA, free from genomic DNA, was isolated using the RNeasy Plus Mini Kit (Qiagen 74134). cDNA was prepared using the SuperScript VILO kit (Invitrogen 11754050).

RNA samples from three representative WT and three E16 livers were selected for RNA-seq analysis. To retain the largest possible messenger RNA complement, samples were subjected to ribosomal RNA depletion rather than polyA^+^ selection. To capture rare events, span splicing junctions, and identify potentially complementary transcripts, over 50 million sequencing reads were acquired in directional, paired-end 2 × 150 format. RNA sequencing operations were done in an Illumina Platform at the Parque Científico de Madrid-UAM (Spain).

The mouse assembly mm10 was used as reference genome for read alignment. Sequences were aligned with TopHat2 software (RRID:SCR_013035) with default settings, which resulted in > 10^8^ mapped reads per sample (left + right ends). The normalized read counts per gene were used as input for the Bioconductor package edgeR (RRID:SCR_012802) to identify differentially expressed genes between WT and E16 samples, including corrections for read length bias.

To identify differential splicing between WT and E16, transcriptional readthrough events and potential base-compositional biases in exon usage, in-house codes were used as described [23, 25]. Briefly, the number of times each exon was either skipped or included in E16 *vs*. WT Tophat2 junction files were counted and divided by the expression level of the corresponding gene. Similarly, the accuracy of transcription termination was analyzed by counting the number of reads along a 275 basepair window downstream of each polyadenylation site as defined in the PolyASite database [45], and correcting them for the expression level of the corresponding gene. Welch´s t-test was used to determine statistical significance in the splicing and transcription termination differences between E16 and WT. A p-value of 0.05 was used as significance threshold; no FDR was used due to the conservative nature of Welch´s t-test. To analyze potential influences of base composition biases on exon usage, a 250 basepair window was established upstream and downstream RefSeq-defined exon boundaries. For each window, the normalized frequency of all possible combinations of 5-mer motifs (1024) was calculated across all samples, and their Spearman correlation with exon inclusion or exclusion in E16 *vs*. WT, was calculated.

To determine enrichment of coordinately expressed gene sets in E16 *vs*. WT conditions, and to connect these sets to known gene ontologies, GSEA (version 4.2.3; RRID:SCR_003199) was applied to RNA-seq datasets. Normalized read counts per gene were compared between E16 and WT livers with default settings. A FDR of 0.1 was used as threshold. For the enrichment analysis of the alternative splicing dataset, exons were first mapped to the corresponding genes. No preselection was carried out; the entire gene complement (20,404 individual genes) was parsed. Ontology terms (hallmark H section for pathways) were identified from the molecular signatures database (MSigDB) version 7.5.1 (RRID:SCR_016863).

Schematic views of selected loci, coverage, and read alignments were produced using IGV [46].

## Supplementary Information

**Additional file 1: **Hepatitis remission in E16 mice.

A**Additional file 2**: Overview of tissues other than liver.

**Additional file 3**: Video of a representative DIDO3-deficient mouse unsteady on the cage wires.

**Additional file 4**: List of genes over- or underexpressed in E16 vs. WT livers.

**Additional file 5**: Analysis of exon usage in E16 vs. WT livers.

**Additional file 6**: Analysis of PAS usage in E16 vs. WT livers.

**Additional file 7**: Analysis of compositional biases around splice sites in E16 vs. WT livers.

**Additional file 8**: Genetic structure of mRNA targets selected for qPCR.

**Additional file 9**: List of oligonucleotide primers.

## Data Availability

Sequencing data are registered with the BioProject database with ID: PRJNA768978. Python 2.7 scripts used to generate data are available from https://github.com/kvanwely/DIDO-LAB. The relevant mouse strain is available under a Material Transfer Agreement.
