# Correction to: Hepatitis, testicular degeneration, and ataxia in DIDO3-deficient mice with altered mRNA processing

**DOI:** 10.1186/s13578-022-00843-1

**Published:** 2022-09-02

**Authors:** Julio Gutiérrez, Karel H. M. van Wely, Carlos Martínez-A

**Affiliations:** Department of Immunology and Oncology, Centro Nacional de Biotecnología-CSIC, Darwin 3, 28049 Madrid, Spain

## Correction to: Cell & Bioscience (2022) 12:84 https://doi.org/10.1186/s13578-022-00804-8

After publication of the original article [1], we realized that two files in the Supplementary Information, Additional files 4 and 7, were incorrect. The correct Additional files 4 and 7 are available on *Cell & Bioscience*’s website from the date of publication of this note.

In addition, a part of the Methods section was missing in the original article. Methods for Additional file 4 are as follows:

Burrows-Wheeler aligner BWA-MEM 0.7.15 (RRID:SCR_010910) was used to align paired-end reads to the UCSC mouse genome build mm10 with standard settings. Alignments were converted to BAM format and de-duplicated with Picard tools 2.9.0 (RRID:SCR_006525). To quantify relative expression of transcripts, we ran StringTie 1.3.3 (RRID:SCR_016323) [2] and calculated the transcripts per million (TPM) reads. Sample scaling and statistical analyses were performed with the R package edgeR (RRID:SCR_012802) [3]. Transcripts with TPM > 0 in all samples were kept for downstream analysis. Differentially expressed genes with an absolute value of log2 fold change ≥ 1 and a false-discovery rate (FDR) < 0.05 were considered statistically significant.

## Supplementary Information

**Additional file 4.** List of genes over- or underexpressed in E16 vs. WT livers.

**Additional file 7.** Analysis of compositional biases around splice sites in E16 vs. WT livers.
