# Supplementary material for: Hepatitis, testicular degeneration, and ataxia in DIDO3-deficient mice with altered mRNA processing
Source: Cell Biosci. 2022 Jun 7;12:84. doi: 10.1186/s13578-022-00804-8 (PMC9172153; doi:10.1186/s13578-022-00804-8)
Supplement: Supplementary file 1 — Additional file 1: Hepatitis remission in E16 mice. [file 13578_2022_804_MOESM1_ESM.docx]

**Additional files (Part). Cell & Bioscience**

**Hepatitis, testicular degeneration and ataxia in DIDO3-deficient mice with altered mRNA processing**

Julio Gutiérrez*, Karel H. M. van Wely and Carlos Martínez-A.

Department of Immunology and Oncology, Centro Nacional de Biotecnología-CSIC Darwin 3, 28049 Madrid, Spain

*Corresponding author: jgutierrez@cnb.csic.es


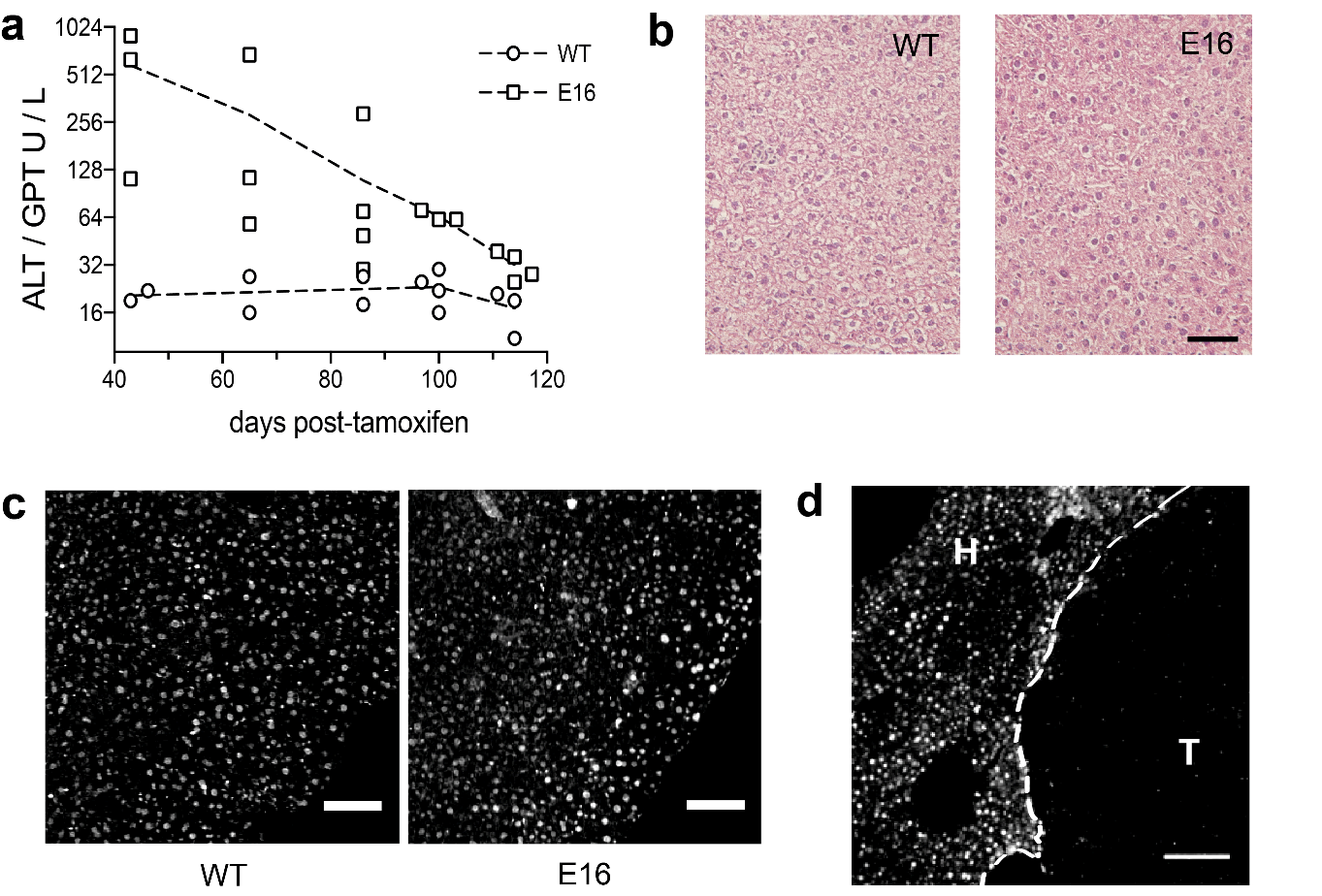


**Additional file 1** Hepatitis remission in E16 mice**. a** Late time course of alanine aminotransferase activity in serum samples from *Dido1* WT (n = 13) and E16 (n = 18) mice. Each mouse was bled only once. Individual values as well as dashed trend lines along means are shown. Aspartate aminotransferase activity followed a similar course. **b** Hematoxylin and Eosin-stained sections of liver samples from representative WT and E16 mice at late stages. Bar = 100 μm. **c** Anti-DIDO3 fluorescent staining of liver sections at late stages. Bar = 100 μm. **d** Anti-DIDO3 fluorescent staining of the transition between healthy (H) and tumor (T) tissues in a liver section from a representative old E16 mouse. A dashed line depicts the boundary. Empty spaces in healthy tissue are vessel sections. Bar = 150 μm
