# Supplementary material for: Hepatitis, testicular degeneration, and ataxia in DIDO3-deficient mice with altered mRNA processing
Source: Cell Biosci. 2022 Jun 7;12:84. doi: 10.1186/s13578-022-00804-8 (PMC9172153; doi:10.1186/s13578-022-00804-8)
Supplement: Supplementary file 2 — AAdditional file 2: Overview of tissues other than liver. [file 13578_2022_804_MOESM2_ESM.docx]

**Additional file 2** Overview of tissues other than liver. **a** Organ / body weight ratios for 2 representative *Dido1* WT and 4 E16 mice; 1 female (F) in each group. **b-g** Hematoxylin and Eosin-stained sections of **b** jejunum, **c** kidney, **d** spleen, **e** heart, **f** lung, **g** brain. **h** Nissl-stained sections of cerebelli. **i** Isolated testes.­­­­­­ Scale bars are depicted in μm
