# Supplementary material for: Hepatitis, testicular degeneration, and ataxia in DIDO3-deficient mice with altered mRNA processing
Source: Cell Biosci. 2022 Jun 7;12:84. doi: 10.1186/s13578-022-00804-8 (PMC9172153; doi:10.1186/s13578-022-00804-8)
Supplement: Supplementary file 8 — Additional file 8: Genetic structure of mRNA targets selected for qPCR. [file 13578_2022_804_MOESM8_ESM.docx]

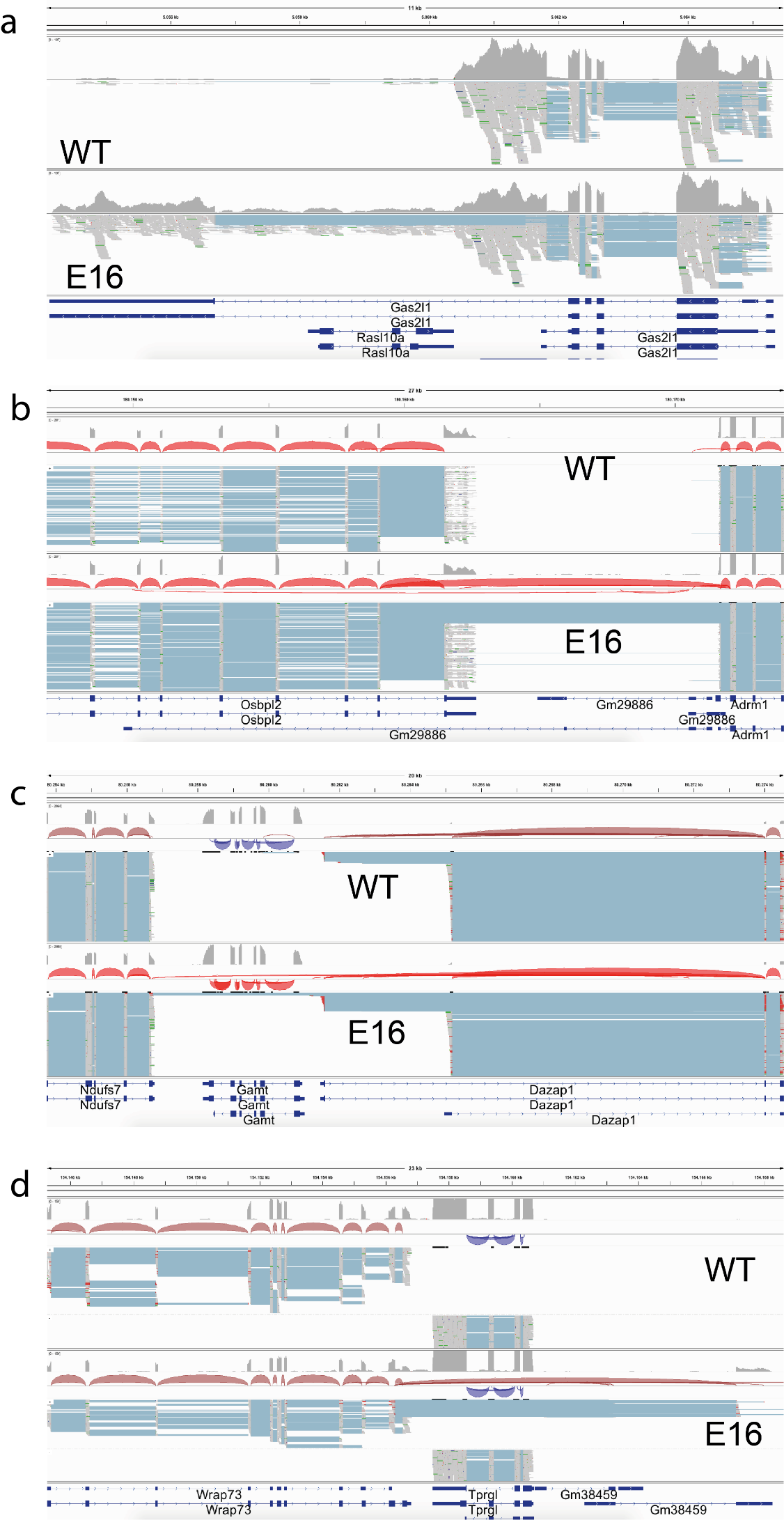


**Additional file 8** Genetic structure of mRNA targets selected for qPCR. Coverage and alignment of RNAseq reads from representative *Dido1* WT and E16 livers to the mm10 reference genome at the following loci: **a** *Gas2L1* (- strand); usage of a distal terminal exon with partial retention of the corresponding intron. **b** *Osbpl2* (+ strand)**;** skipping of its last exon, transcriptional readthrough and aberrant splicing of the penultimate exon into the following gene in the same strand, *Adrm1*. **c** A similar transcriptional jump, 17 kb long, from the last exon of *Ndufs7* to the third exon of the following gene, *Dazap1* (both + strand). **d** *Wrap73* (+ strand) similarly joined to an otherwise silent *Gm38459*, over heavily expressed *Tprgl* in the - strand. For clarity, splice junction tracks are shown in b, c and d
