# Supplementary material for: Hepatitis, testicular degeneration, and ataxia in DIDO3-deficient mice with altered mRNA processing
Source: Cell Biosci. 2022 Jun 7;12:84. doi: 10.1186/s13578-022-00804-8 (PMC9172153; doi:10.1186/s13578-022-00804-8)
Supplement: Supplementary file 9 — Additional file 9: List of oligonucleotide primers. [file 13578_2022_804_MOESM9_ESM.docx]

**Additional File 9** List of oligonucleotide primers
